# Arginine-substituted Mastoparan-C derivatives combat dual bacterial pathogens: *in vitro* mechanistic insights and *in vivo* efficacy in polymicrobial wounds

**DOI:** 10.1128/spectrum.04219-25

**Published:** 2026-06-29

**Authors:** Luyao Zhang, Anqi Huang, Mingyu Lu, Jingjing Wang, Yingyu Wang, Fei Mo, Mingchun Liu

**Affiliations:** 1 College of Animal Science and Medicine, Shenyang Agricultural University, Shenyang, Liaoning, China; Zhejiang University School of Medicine Sir Run Run Shaw Hospital, Zhejiang, China

**Keywords:** antimicrobial peptides, wound polymicrobial infection, structure-activity relationship, arginine modification, membrane disruption

## Abstract

The synergistic interactions in multi-pathogen infections compromise wound healing and limit therapeutic efficacy. In this study, we designed and synthesized arginine-substituted derivatives of the antimicrobial peptide Mastoparan-C (MP-C). Among them, Arg²MP-C and Arg^4.11.12^MP-C exhibited potent, broad-spectrum activity against both *Escherichia coli* and *Staphylococcus aureus*. Their enhanced antibacterial activity is associated with increased positive charge and optimized hydrophobicity. Mechanistically, both peptides employ a dual-target strategy, disrupting bacterial membranes and binding genomic DNA; Arg²MP-C acted most rapidly against the *E. coli* envelope, while Arg^4.11.12^MP-C caused the strongest membrane damage to *S. aureus*. In a murine polymicrobial wound model, Arg²MP-C treatment nearly achieved complete wound closure by day 10, significantly reduced bacterial loads, and promoted tissue regeneration. This study demonstrates that arginine engineering can yield peptides with potent, multi-mechanistic action, identifying Arg²MP-C as a promising candidate for combating polymicrobial wound infections.

**IMPORTANCE:**

Wounds infected with multiple bacterial species are notoriously difficult to treat, often leading to poor healing and limited effectiveness of existing therapies. In this study, we developed new antimicrobial peptides by introducing arginine substitutions into a natural peptide called Mastoparan-C. Two of our engineered peptides, Arg²MP-C and Arg^4.11.12^MP-C, showed potent activity against two common wound pathogens, *Escherichia coli* and *Staphylococcus aureus*. These peptides work through a dual mechanism: disrupting bacterial membranes and binding to bacterial DNA. In a mouse model of mixed-infection wounds, treatment with Arg²MP-C led to nearly complete wound closure by day 10, drastically reduced bacterial counts, and promoted tissue repair. Our findings highlight arginine engineering as a promising strategy to create next‑generation antimicrobial agents that can effectively combat complex polymicrobial wound infections, addressing a critical unmet need in clinical wound care.

## INTRODUCTION

Wound infection is an inflammatory response of the host to microorganisms that can destroy host tissues and thus impede wound healing (1). Bacterial infections, a major complication of skin trauma characterized by high recurrence rates and slow healing, are often driven by pathogens expressing specialized virulence factors for adhesion and invasion (2). Critically, the wound microbiota modulates repair processes and infection susceptibility through dynamic interactions with bacterial biofilms (3, 4). In polymicrobial environments, interspecies synergies or antagonisms further perturb biofilm dynamics and therapeutic outcomes (5). The infection of bacteria at the wound site can delay wound healing, lead to chronicity of the wound, and increase the risk of hospitalization, among other consequences. Due to the aging population and the increasing incidence of diabetes, obesity, and systemic diseases, chronic wound non-healing has become a growing challenge worldwide (6).

The primary treatment strategy for polymicrobial wounds involves debridement, followed by the application of wound dressings, incorporating antimicrobial agents such as antibiotics, natural antimicrobials, nanomaterials, surfactants, photodynamic substances, or nanoparticles (7–10). However, biofilm formation presents a major clinical challenge, as biofilms exhibit significant resistance to conventional antimicrobial treatments (11, 12). Consequently, the recalcitrant nature of chronic wound infections necessitates the urgent development of novel and effective antimicrobial therapies capable of combating polymicrobial infections.

Antimicrobial peptides (AMPs), a class of naturally occurring small proteins or peptides, constitute the first line of defense in organisms (13). As essential components of innate immunity across humans, animals, insects, and plants, they exhibit multifaceted bioactivities beyond direct antimicrobial effects. These include immunomodulation, anti-inflammatory responses, promotion of epithelial cell migration, and angiogenesis—properties that highlight their therapeutic potential for wound infection management (14–17). Notably, AMP-based strategies have shown significant progress in treating skin infections, particularly chronic drug-resistant wounds, such as diabetic ulcers (18, 19). Clinical trials further support their efficacy, with certain AMPs outperforming conventional treatments in skin wound healing (20, 21).

Mastoparans (MPs), a class of cationic AMPs isolated from wasp venoms, exhibit diverse biological activities, including potent broad-spectrum antimicrobial effects against bacteria and fungi (22). Specific variants demonstrate significant therapeutic potential: Polybia-MPII (*Pseudopolybia vespiceps*) combats *in vivo S. aureus* infections (23); Mastoparan X effectively targets MRSA (24); and Mastoparan (*Vespula lewisii*) activates mast cells via MRGPRX2 to enhance the clearance of *S. aureus* skin infections (25). These properties position Mastoparans as promising candidates for wound infection therapy. However, their clinical translation is limited by inherent cytotoxicity and hemolytic activity, challenges common to many natural AMPs. Consequently, structural modification strategies are essential to mitigate adverse effects and elucidate structure-function relationships (26). Promisingly, rationally designed analogs like [I^5^, R^8^]MP (based on Mastoparan-L) demonstrate enhanced selectivity and antibacterial efficacy (27). Similarly, MP9-10 (derived from Mastoparan via tryptophan substitution) exhibits superior activity against multidrug-resistant bacteria with significantly reduced cytotoxicity and hemolysis, highlighting its potential for treating challenging infections like those caused by *Staphylococcus aureus* (28). Therefore, structurally modified MPs represent a promising avenue for developing novel therapeutics against challenging wound infections, particularly those involving drug-resistant bacteria.

Herein, we constructed five amino acid-substituted derivatives based on MP-C, with arginine-modified analogs Arg^2^MP-C and Arg^4.11.12^MP-C demonstrating potent activity against dual bacterial pathogens (*Escherichia coli* and *S. aureus*). These derivatives exhibit enhanced antimicrobial efficacy coupled with reduced cytotoxicity and hemolysis, governed by critical structure-activity relationships (SARs) where increased cationic charge and optimized hydrophobicity improve selectivity. Mechanistic studies reveal their novel dual-target action: simultaneous disruption of bacterial membranes and binding to genomic DNA in both gram-negative and gram-positive pathogens. Crucially, in polymicrobial wound infections, Arg^2^MP-C promoted near-complete tissue regeneration by day 10, significantly reducing bacterial burden. This work establishes arginine-engineered MP-C derivatives as effective dual-pathogen therapeutics, providing a mechanistic blueprint for combating complex wound infections through rational peptide design.

## MATERIALS AND METHODS

### Materials

*E. coli* (ATCC 25922), *S. aureus* (ATCC 29213), and RAW264.7 were purchased from the American Type Culture Collection and maintained in our laboratory. *E. coli* (CICC 10389) was purchased from Beina Chuanglian Biotechnology Co., Ltd., China. MH(A), MH(B), ceftiofur sodium, and Triton X-100 were purchased from Beijing Solarbio Science & Technology Co., Ltd., China. Gentamicin, tylosin, and enrofloxacin were obtained from Dalian Meilun Biotechnology Co., Ltd., China. Fetal bovine serum (FBS), Sytox green dye and Syto9, and propidium iodide (PI) fluorescent dye mixture were obtained from Thermo Fisher Scientific, USA. Crystal violet was supplied by Changde Beckman Biotechnology Co., Ltd., China. PBS was purchased from Wuhan Servicebio Technology Co., Ltd., China. N-phenyl-1-naphthylamine (NPN) was obtained from Shanghai Macklin Biochemical Co., Ltd., China. MTT and o-nitrophenyl β-D-galactopyranoside (ONPG) were obtained from Shanghai Yuanye Bio-Technology Co., Ltd., China; 2.5% glutaraldehyde solution was supplied by Sigma-Aldrich, USA. TIANGEN Bacterial Genomic DNA Extraction Kit was obtained from Tiangen Biotech (Beijing) Co., Ltd., China.

### Design and synthesis of MP-C and its derivatives

The physicochemical parameters of MP-C and its derivatives were predicted using the Heliquest web server (29), and the derivatives were synthesized using solid-phase peptide synthesis techniques. The preparative reversed-phase high-performance liquid chromatography (RP-HPLC, LC-10A, SHIMADZU, Japan) was used for purification, and the mass spectrometer (watersZQ2000, Waters, USA) was used to identify the components of each chromatographic peak. The target drug fractions (MP-C, Pro^5^MP-C, Ile^6^MP-C, Val^10^MP-C, Arg^2^MP-C, and Arg^4,11,12^MP-C) were collected and lyophilized.

### Antibacterial activity

#### Determination of the minimal inhibitory concentrations (MICs)

MICs of MP-C and its derivatives against *E. coli* and *S. aureus* were determined by the broth microdilution method in 96-well plates. Briefly, *E. coli* and *S. aureus* were incubated with different concentrations (128 μM ~ 0.25 µM) of MP-C and its derivatives in MH(B) medium at 37°C for 16–20 h. MICs were the lowest concentrations of MP-C and its derivatives corresponding to the optically clear wells in 96-well plates.

#### Determination of the minimal bactericidal concentrations (MBCs)

In total, 20 μL of the mixture from each optically clear well of the above 96-well plates was aspirated and spread onto the MH(A) plates. After incubation at 37°C for 16–20 h, the growth of bacterial colonies was observed. The lowest concentrations at which no bacterial colonies formed were determined as MBCs of MP-C and its derivatives.

### Checkerboard assay

The checkerboard assay was used to evaluate the antibacterial activity of MP-C and its derivatives in combination with four conventional antibiotics against *E. coli* and *S. aureus*. Both agents were serially diluted to concentrations ranging from 0.0625× MIC to 2× MIC. After incubation with bacterial suspensions at 37°C for 18–24 h, the fractional inhibitory concentration indices were calculated.

### Anti-biofilm activity

*E. coli* and *S. aureus* were separately incubated with different concentrations of MP-C and its derivatives in MH(B) medium supplemented with 8% FBS at 37°C under static (non-shaking) conditions for 24 h. The inhibition of biofilm formation by each bacterium was then assessed using the crystal violet staining method. Furthermore, suspensions of *E. coli* and *S. aureus* were mixed at a 1:1 ratio, seeded into 96-well plates, and co-incubated with various concentrations of each peptide at 37°C under static conditions for 48 h, with blank medium and growth control groups also included. The crystal violet staining method was similarly employed to evaluate the inhibitory effect on the dual-species mixed biofilm. The minimum biofilm inhibitory concentrations (MBICs) were defined as the lowest concentrations of MP-C and its derivatives at which a further increase in concentration led to no significant change in the biofilm inhibition rate.

### Safety study

#### Cytotoxicity

The cytotoxicity of MP-C and its derivatives against mouse mononuclear macrophages RAW264.7 was evaluated by MTT assay. Briefly, RAW264.7 cells were seeded in a 96-well plate with a density of 7,500 cells/well. Then, different concentrations of MP-C and its derivatives were added into wells. After incubation at 37°C for 24 h, the MTT assay was conducted, and the absorbance values at 550 nm were measured by a microplate spectrophotometer system (VICTOR Nivo, PerkinElmer, Inc., USA) to determine cell viability.

#### Hemolysis assessment

In total, 2% red blood cell solution was prepared using rabbit blood. Different concentrations of MP-C and its derivatives were mixed with 2% red blood cell solution in equal volumes. After incubation at 37°C for 2 h, the mixture was centrifuged at 2,500 rpm for 5 min. The absorbance of the supernatant at 550 nm was measured by a microplate spectrophotometer system (VICTOR Nivo, PerkinElmer, Inc., USA), and the hemolysis rate (%) was calculated; 2% Triton X-100 and PBS were served as the positive control and the negative control, respectively.

### Cell membrane permeability assay of *E. coli* and *S. aureus*

#### Outer and inner membrane permeability assay of *E. coli*

*E. coli* in the logarithmic growth phase was diluted to OD_600_ = 0.5. Different concentrations of MP-C and its derivatives were mixed with the fluorescent dye NPN (40 μM) in a light-protected 96-well plate. Subsequently, 100 μL of *E. coli* suspension was added into each well and mixed thoroughly. The fluorescence intensity was immediately measured using a microplate spectrophotometer system (VICTOR Nivo, PerkinElmer, Inc., USA) for 15 min, with an excitation wavelength of 350 nm and an emission wavelength of 420 nm. PBS was used as a blank control.

The isolate *E. coli* CICC 10389 was cultured in MH(B) medium supplemented with 5% lactose to the logarithmic growth phase and then diluted to OD_420_ = 1.2. Different concentrations of MP-C and its derivatives were mixed with substrate ONPG (30 mM) in a 96-well plate. *E. coli* suspension was added, and the absorbance at 420 nm was monitored using a microplate spectrophotometer system (VICTOR Nivo, PerkinElmer, Inc., USA) for 140 min. PBS was used as a blank control.

#### Cell membrane disruption assay of *S. aureus*

*S. aureus* in the logarithmic growth phase was diluted to OD_600_ = 0.2 and mixed with Sytox green dye (0.3 μM). The fluorescence intensity was monitored (excitation wavelength and emission wavelength were 504 nm and 523 nm) until a stable baseline signal was achieved. Subsequently, peptide solutions at different concentrations were added to *S. aureus* suspension, and the changes in fluorescence intensity were recorded continuously for 1 h. Besides, *S. aureus* was diluted to 1 × 10^8^ CFU/mL, treated with different concentrations of peptides, and incubated at 37°C for 45 min. Subsequently, *S. aureus* suspensions were stained with Syto9 (5 μM) and PI (30 μM), followed by incubation in the dark for 20 min. The mixtures were then centrifuged to remove excess dyes, resuspended, and observed under an inverted fluorescence microscope (Primovert, Zeiss, Germany). Quantitative analysis was subsequently performed using ImageJ. PBS served as the blank control in the above experiments.

### Morphological study of *E. coli* and *S. aureus*

*E. coli* and *S. aureus* in the logarithmic growth phase were diluted to 1 × 10⁸ CFU/mL, respectively. Subsequently, the antimicrobial agents (MP-C, Arg^2^MP-C, and ceftiofur sodium) were diluted to 4× MIC and added to the bacterial suspensions at a 1:100 (vol/vol) ratio. After incubation at 37°C for 45 min, the samples were fixed overnight with 2.5% glutaraldehyde solution at 4°C. Finally, the samples were prepared according to standard SEM (Regulus 8100, Hitachi, Japan) protocols and observed.

### Electrophoretic mobility shift assay

Genomic DNA of *E. coli* and *S. aureus* was extracted using the TIANGEN Bacterial Genomic DNA Extraction Kit according to the manufacturer’s instructions. Following quantification with a Nanodrop spectrophotometer, 30 μL of the genomic DNA was incubated with a series of concentrations of MP-C, Arg^2^MP-C, and Arg^4.11.12^MP-C for 30 min. The resulting samples were analyzed by electrophoresis on a 1% agarose gel.

### Animal experiment

Female Kunming SPF-grade mice were randomly assigned to six experimental groups: MP-C group, Arg^2^MP-C group, Arg^4.11.12^MP-C group, model group, positive drug control (ciprofloxacin) group, and normal group. Following anesthesia, the dorsal skin of the mouse was surgically excised symmetrically to create full-thickness wounds (0.8 cm× 0.8 cm) under aseptic conditions. The wounds were inoculated with 100 μL of a mixed bacterial suspension of *E. coli* and *S. aureus* (1:1 ratio) at 5 × 10^7^ CFU/mL. The inoculation was repeated once after 3 h, for a total inoculation volume of 200 μL. After 2 consecutive days of model establishment, the wound status was observed on day 3. To confirm successful modeling, pus samples were collected and cultured on MacConkey agar for *E. coli* and mannitol salt agar for *S. aureus* (30, 31).

Mice with confirmed infections received daily topical applications of 12 μg/mL of each of three peptides (MP-C, Arg^2^MP-C, or Arg^4.11.12^MP-C) or 1 μg/mL ciprofloxacin. Wound healing was assessed every 48 h, with the healing rate calculated (equation 1) and a healing rate curve plotted (32). After 10 days of treatment, all mice were euthanized by CO_2_ asphyxiation. Eschar tissues from the wound sites were harvested for histopathological analysis via hematoxylin-eosin (HE) staining, which was performed by Shenyang Shengqiyuan Biotechnology Co., Ltd. Additionally, wound tissue samples were homogenized and cultured on the same selective media to quantify the bacterial burden.


(1) $$\text{Healing rate}\left(\%\right)\text{=}\frac{\text{Original wound area-current wound area}}{\text{Original wound area}}\times 100\%$$

### Statistical analysis

All experiments were performed in at least three independent biological replicates, and data are presented as mean ± standard deviation (SD). Comparisons among multiple groups were analyzed by one-way or two-way analysis of variance followed by Tukey’s post-hoc test. Statistical significance was set at *P* < 0.05 (*) and *P* < 0.01 (**), indicating significant and highly significant differences, respectively.

## RESULTS

### Design and synthesis of MP-C and its derivatives

Structural properties of MP-C and its derivatives were summarized in Table 1. Net charges ranged from +4 to +5, with hydrophobicity elevated in all variants except two arginine-substituted derivatives. Hydrophobic moments exhibited irregular changes. Mass spectrometry verified molecular masses (Fig. S1), while reversed-phase HPLC confirmed >90% purity for all peptides (Fig. S2).

**TABLE 1:** Structural properties of MP-C and its derivatives

Peptides	Sequence	Hydrophobicity (H）	Hydrophobic moment (μH）	Net charge
MP-C	LNLKALLAVAKKIL-NH_2_	0.634	0.399	+4
Pro^5^MP-C	LNLKPLLAVAKKIL-NH_2_	0.664	0.384	+4
Val^10^MP-C	LNLKALLAVVKKIL-NH_2_	0.699	0.461	+4
Ile^6^MP-C	LNLKAILAVAKKIL-NH_2_	0.641	0.405	+4
Arg^2^MP-C	LRLKALLAVAKKIL-NH_2_	0.605	0.386	+5
Arg^4.11.12^MP-C	LNLRALLAVARRIL-NH_2_	0.630	0.401	+4

### Antibacterial activity

MP-C and its derivatives exhibited potent antibacterial activity against both pathogens (Table 2). The ranges of MIC against *E. coli* and *S. aureus* were 2–4 µM and 1–4 µM, respectively. Notably, compared with MP-C, Ile^6^MP-C and Arg^2^MP-C exhibited enhanced antibacterial activity against *E. coli*, while Arg^4.11.12^MP-C showed superior antibacterial activity against *S. aureus*. Corresponding MBC results further confirmed the strong bactericidal activity of MP-C and its derivatives (Table 2). The MBC values ranged from 4 μM to 32 μM for *E. coli* and from 1 μM to 8 μM for *S. aureus*. Compared with MP-C, Ile^6^MP-C, Arg^2^MP-C, and Arg^4.11.12^MP-C reduced MBCs by 4-fold to 8-fold against *E. coli*, while five derivatives exhibited a 2-fold to 8-fold decrease in MBCs against *S. aureus*.

**TABLE 2:** Antibacterial activity of MP-C and its derivatives against two bacterial strains

	MIC/MBC (μM)					
	MP-C	Pro^5^MP-C	Val^10^MP-C	Ile^6^MP-C	Arg^2^MP-C	Arg^4.11.12^MP-C
*E. coli*	4/32	4/32	4/32	2/8	2/4	4/8
*S. aureus*	2/8	2/4	2/2	4/4	2/2	1/1

**Fig 1:**
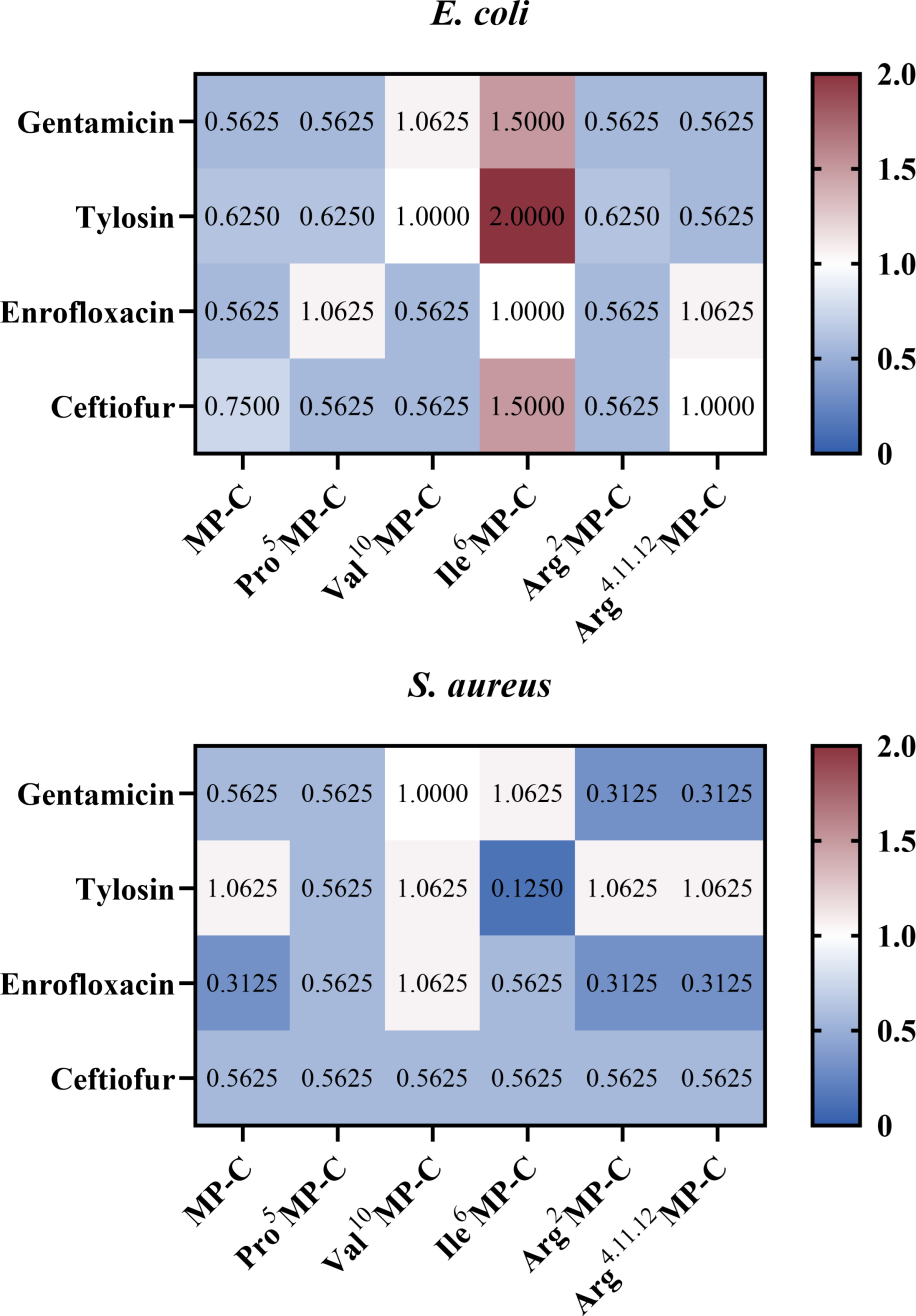
Checkerboard assay results for MP-C and its derivatives against *E. coli* and *S. aureus*. The heatmaps show the synergistic effects of combinations with conventional antibiotics.

The results of the checkerboard assay were shown in Fig. 1 and Table S1. Against *E. coli*, most combinations exhibited additive effects (15 of 24). Notably, the parent peptide MP-C and Arg^2^MP-C showed additive effects with all four antibiotics. Pro^5^MP-C showed additive effects with most antibiotics, except for enrofloxacin, which showed indifferent effects. Val^10^MP-C and Arg^4.11.12^MP-C exhibited additive effects with two antibiotics and indifferent effects with the other two antibiotics. However, Ile^6^MP-C displayed indifferent effects with all antibiotics, suggesting that this derivative might not enhance the activity of conventional antibiotics against *E. coli*. Against *S. aureus*, more synergistic effects were observed (6 of 24). Arg^2^MP-C and Arg^4.11.12^MP-C showed synergistic effects with two antibiotics, and MP-C and Ile^6^MP-C showed synergistic effects with one antibiotic.

Collectively, these results indicated that specific amino acid substitutions significantly influence the antibacterial activity of MP-C. The differential effects against gram-negative (*E. coli*) and gram-positive (*S. aureus*) bacteria might reflect variations in cell wall structure and peptide penetration efficiency.

### Anti-biofilm activity

#### Mono-species

As summarized in Table 3, MP-C and its derivatives showed lower MBIC values against *S. aureus* than against *E. coli*, indicating a substantially stronger ability to inhibit biofilm formation of *S. aureus*. Notably, Arg^2^MP-C and Arg^4.11.12^MP-C exhibited a 4-fold reduction in MBIC against *E. coli* and a 2-fold reduction against *S. aureus* compared with MP-C. These results suggested that specific amino acid substitutions of MP-C could effectively improve the anti-biofilm activity against monospecies *E. coli* and *S. aureus*.

**TABLE 3:** Anti-biofilm activity of MP-C and its derivatives against monospecies and dual species^*a*^

	MBIC (μM)					
	MP-C	Pro^5^MP-C	Val^10^MP-C	Ile^6^MP-C	Arg^2^MP-C	Arg^4.11.12^MP-C
*E. coli*	64	64	64	64	16	16
*S. aureus*	8	16	16	16	4	4
Mixed	64	NT	NT	NT	32	32

^*a*^ NT, not tested.

### Dual-species

Inhibition assays of MP-C and its derivatives against dual-species mixed biofilms of *E. coli* and *S. aureus* showed that the MBIC values of MP-C, Arg^2^MP-C, and Arg^4.11.12^MP-C were 64, 32, and 32 μM, respectively (Table 3). The other three derivatives were not evaluated in the mixed biofilm assay. These results indicated that specific amino acid substitutions of MP-C could also enhance its inhibitory effect against dual-species mixed biofilms.

### Safety study

**Fig 2:**
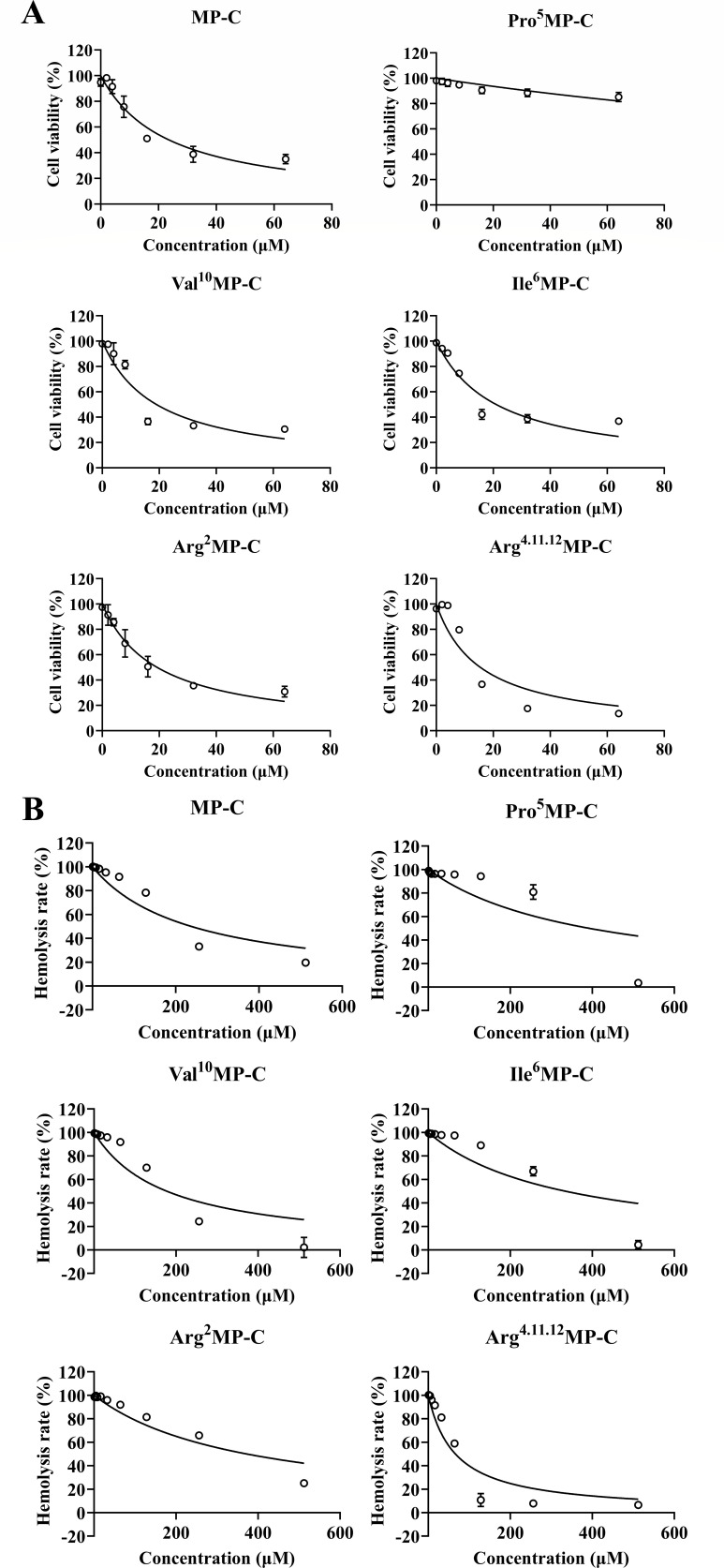
Cytotoxicity and hemolytic activity of MP-C and its derivatives. (**A**) Cytotoxicity against mouse mononuclear macrophages RAW264.7. (**B**) Hemolytic activity against rabbit red blood cells.

The results of the *in vitro* safety evaluation are shown in Fig. 2A and B. MP-C and its derivatives induced concentration-dependent cytotoxicity in RAW264.7 macrophages (Fig. 2A). The IC_50_ values of MP-C, Pro^5^MP-C, Val^10^MP-C, Ile^6^MP-C, Arg^2^MP-C, and Arg^4.11.12^MP-C were 23.59 ± 2.18, 291.4 ± 32.40, 19.01 ± 2.39, 21.02 ± 2.13, 19.26 ± 1.55, and 15.48 ± 2.46 µM, respectively. Compared with MP-C, Pro^5^MP-C showed significantly lower cytotoxicity (*P* < 0.01), whereas Arg^2^MP-C (*P* < 0.05) and Arg^4.11.12^MP-C (*P* < 0.01) exhibited significantly higher cytotoxicity. No significant difference in cytotoxicity was observed for Val^10^MP-C and Ile^6^MP-C relative to MP-C.

In the hemolysis assay, the HC_50_ values of MP-C, Pro^5^MP-C, Val^10^MP-C, Ile^6^MP-C, Arg^2^MP-C, and Arg^4.11.12^MP-C were 237.3 ± 27.72, 392.1 ± 87.76, 177.2 ± 27.67, 334.1 ± 62.63, 372.3 ± 35.57, and 66.18 ± 8.85 µM, respectively (Fig. 2B). Compared with MP-C, all five modified peptides showed significant differences in hemolytic activity (*P* < 0.01). Specifically, Val^10^MP-C and Arg^4.11.12^MP-C exhibited enhanced hemolytic activity, while Pro^5^MP-C, Ile^6^MP-C, and Arg^2^MP-C showed a reduction. Combined with the cytotoxicity findings, these results suggested that Pro^5^MP-C had the most favorable safety profile and that amino acid substitutions in MP-C could influence its safety properties.

### Influence of MP-C and its derivatives on the cell membranes of *E. coli* and *S. aureus*

**Fig 3:**
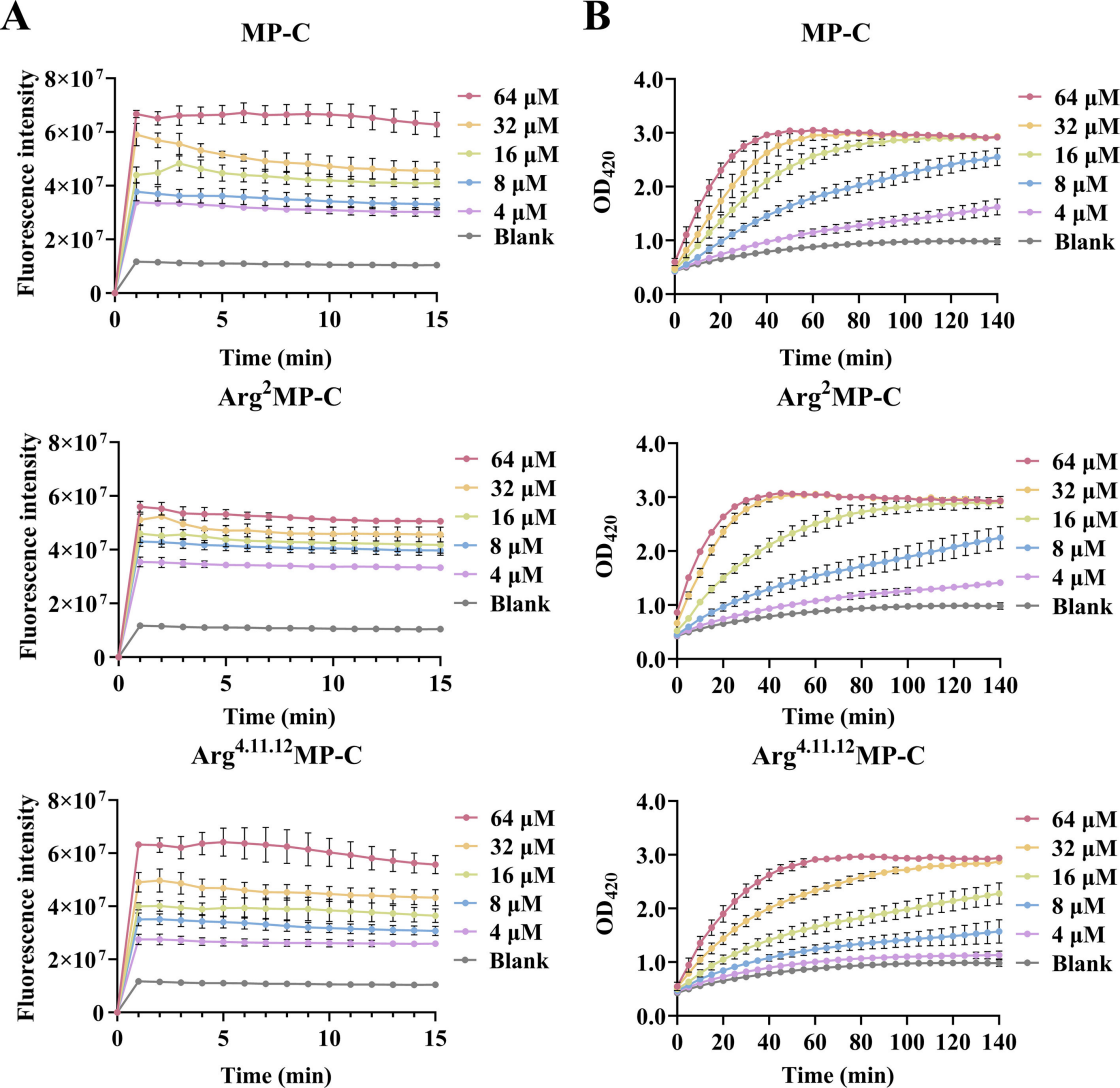
The influence of MP-C and its derivatives on *E. coli* membrane integrity. (**A**) Outer membrane permeability. (**B**) Inner membrane permeability.

As shown in Fig. 3A, MP-C and its derivatives induced a significant increase in fluorescence intensity in a concentration-dependent manner compared to the blank group, demonstrating their disruptive effect on the outer membrane of *E. coli*. Further comparison at 16 μM, the observed fluorescence intensity ranked Arg^2^MP-C >MP C>Arg^4.11.12^MP-C after 1 min of exposure. However, following extended exposure (3 min), the order shifted to MP-C > ArgMP-C > Arg^4.11.12^MP-C. This kinetic profile implied a time-dependent enhancement in the outer membrane disruption caused by MP-C in *E. coli*, whereas the disruptive effects of Arg^2^MP-C and Arg^4.11.12^MP-C remained comparatively constant over the observed duration.

Similarly, disruption of the *E. coli* inner membrane by MP-C and its derivatives showed clear concentration and time dependence (Fig. 3B). Both higher peptide concentrations and longer exposure times led to progressively greater membrane damage. A comparison at 32 μM revealed a consistent rank order of inner membrane disruption efficacy at 5 and 25 min: Arg^2^MP-C  > MP C > Arg^4.11.12^MP-C (Fig. S3). These results demonstrated that Arg^2^MP-C exhibited the fastest onset and highest potency in disrupting the inner membrane of *E. coli*.

**Fig 4:**
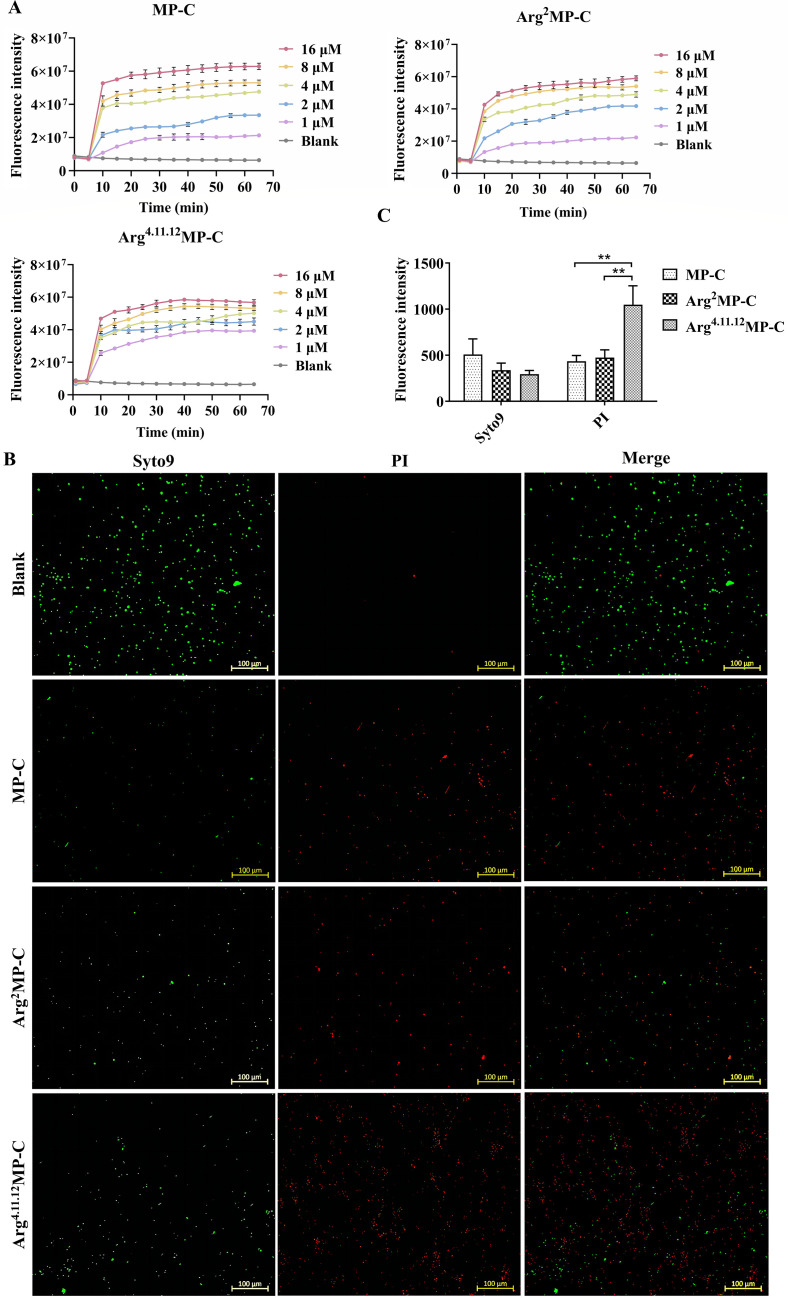
Membrane disruption of *S. aureus* induced by MP-C and its derivatives. (**A**) Sytox green uptake assay. (**B**) Fluorescence microscopy images of *S. aureus* stained with Syto9 and PI. (**C**) Quantitative analysis of fluorescence intensity in (**B**) using Image J. ** indicates highly significant differences (*P* < 0.01).

In the *S. aureus* membrane disruption assay, treatment with MP-C and its derivatives also elicited a time- and concentration-dependent increase in fluorescence intensity relative to the blank control, confirming their membrane-disrupting effects (Fig. 4A). At 8 μM, the extent of membrane damage at 10 min followed the order MP-C > ArgMP-C > Arg^2^MP-C, whereas by 30 min the order shifted to Arg^4.11.12^MP-C > Arg^2^MP-C >MP C. These results indicated that the membrane-disruptive effect of Arg^4.11.12^MP-C against *S. aureus* intensified more substantially over time. Consistent with these findings, fluorescence microscopy observations revealed that compared to the blank control, all three peptide treatment groups exhibited a reduction in green fluorescence and an increase in red fluorescence (Fig. 4B). Quantitative analysis using Image J showed that the red fluorescence intensity in the Arg^4.11.12^MP-C group was significantly higher than that in both the MP-C and Arg^2^MP-C groups, indicating that Arg^4.11.12^MP-C possessed the strongest membrane-disruptive effect against *S. aureus* (Fig. 4C).

### Influence of MP-C and its derivatives on the cellular morphology of *E. coli* and *S. aureus*

**Fig 5:**
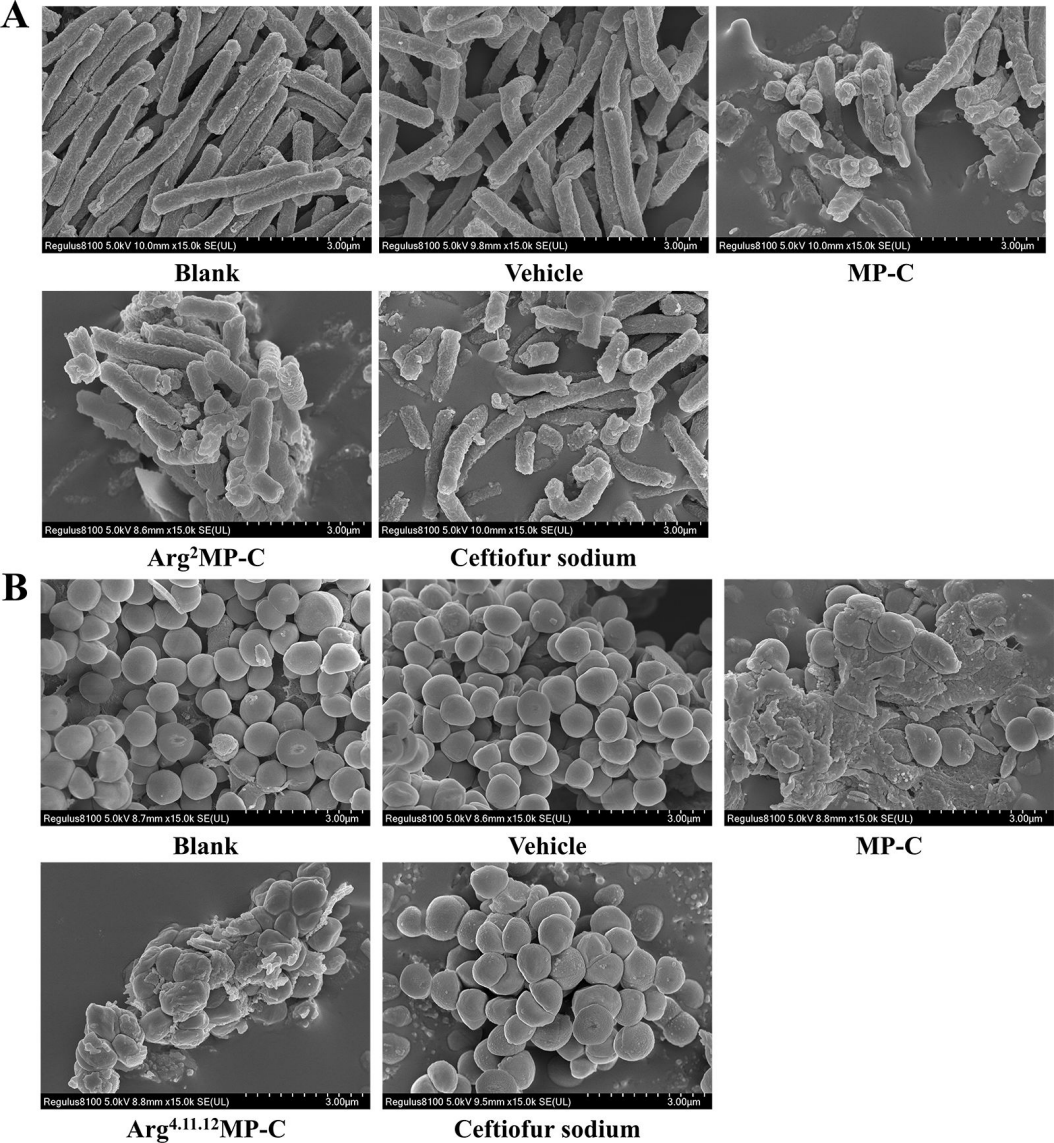
SEM images showing morphological changes of bacteria treated with MP-C and its derivatives (15,000×). (**A**) *E. coli*. (**B**) *S. aureus*.

SEM revealed the morphological changes in *E. coli* and *S. aureus* after treatment (Fig. 5A and B). Untreated (blank and vehicle groups) cells of *E. coli* possessed intact, long-rod structures with smooth surfaces, while *S. aureus* cells were spherical and equally intact. In contrast, treatment with MP-C or its derivatives or ceftiofur sodium induced significant membrane shrinkage and disruption in both bacteria. Notably, MP-C and Arg^4.11.12^MP-C caused more severe morphological damage to *S. aureus* than ceftiofur sodium.

### Genomic DNA targeting validation

**Fig 6:**
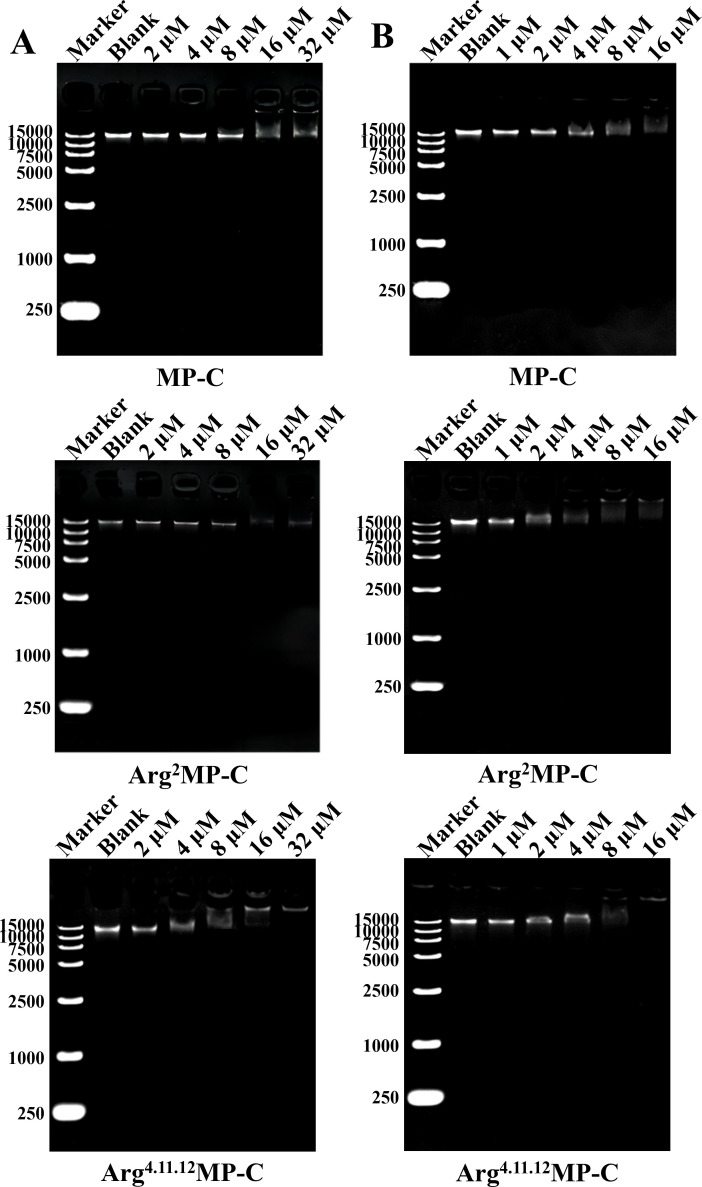
DNA-binding affinity of MP-C and its derivatives. (**A**) Binding to *E. coli* genomic DNA. (**B**) Binding to *S. aureus* genomic DNA.

In the electrophoretic mobility shift assay, the binding of peptides to bacterial genomic DNA was evidenced by a reduction in DNA mobility, manifested as trailing or even complete retention within the agarose gel, with the extent of shift correlating directly with binding affinity. As shown in Fig. 6, treatment with MP-C and its derivatives induced such concentration-dependent shifts in the genomic DNA of both *E. coli* and *S. aureus*, confirming their general DNA-binding capability.

Specifically, for *E. coli* genomic DNA, the onset of detectable retardation occurred at concentrations of 16, 16, and 4 μM for MP-C, Arg^2^MP-C, and Arg^4.11.12^MP-C, respectively, indicating that Arg^4.11.12^MP-C possessed the strongest binding affinity (Fig. 6A). Conversely, for *S. aureus* genomic DNA, retardation was observed starting at lower concentrations of 4, 2, and 2 μM for the three peptides (Fig. 6B). These results demonstrated that specific amino acid substitutions could significantly influence the DNA-binding capability of MP-C.

#### *In vivo* wound healing efficacy

**Fig 7:**
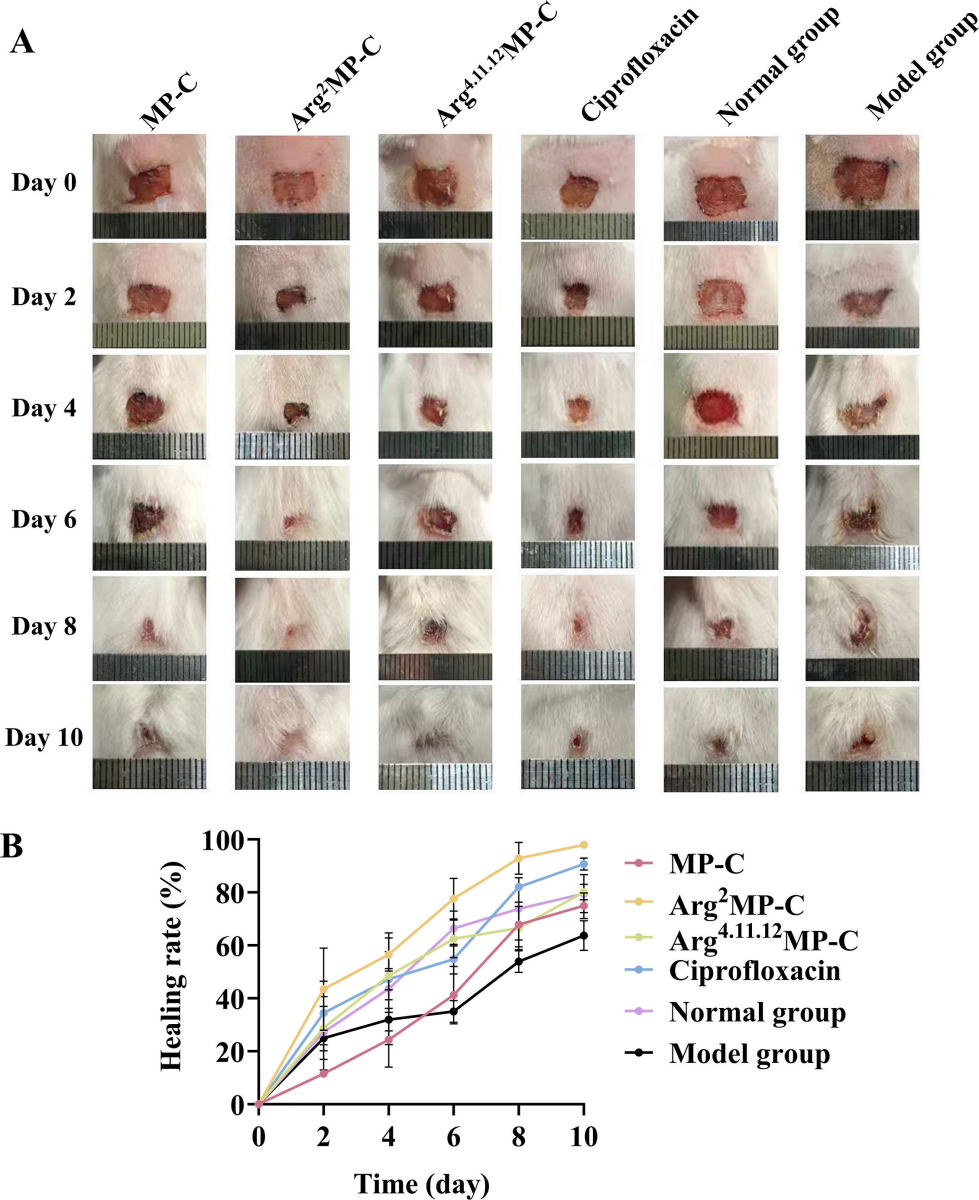
*In vivo* wound healing efficacy of MP-C and its derivatives in a mouse model of skin infection. (**A**) Representative photographs of wounds at indicated time points. (**B**) Healing rate curve of infected skin wounds in mice.

The therapeutic effects of MP-C and its derivatives on skin-infected wounds in mice were shown in Fig. 7A. The wounds in all groups gradually healed over time, with a progressive reduction in wound area. On day 2, no purulent discharge was observed upon applying pressure to the wounds in any treatment group (MP-C, Arg^2^MP-C, Arg^4.11.12^MP-C, and ciprofloxacin group), and a thin layer of scab formed, indicating that these treatments exerted a certain inhibitory effect on wound infection. On day 6, mice in the Arg^2^MP-C group exhibited the most rapid and pronounced wound recovery compared with the other groups. On day 10, the wounds in the Arg^2^MP-C group were almost completely healed.

Quantitative analysis of wound healing in mice using ImageJ demonstrated that on day 6, the wound healing rates of mice treated by MP-C, Arg^2^MP-C, Arg^4.11.12^MP-C, and ciprofloxacin were 41.23% ± 10.82%, 77.59% ± 7.74%, 62.49% ± 7.08%, and 54.78% ± 5.58%, respectively. On day 10, the corresponding healing rates were 74.93% ± 4.85%, 97.93% ± 0.23%, 80.09% ± 2.91%, and 90.72% ± 2.29%. Taken together, these findings suggested that MP-C and its arginine derivatives exhibited therapeutic effects on mice skin wound infections, among which Arg^2^MP-C exerted the most pronounced pro-healing effect (Fig. 7B).

### Histopathological analysis of skin tissue

**Fig 8:**
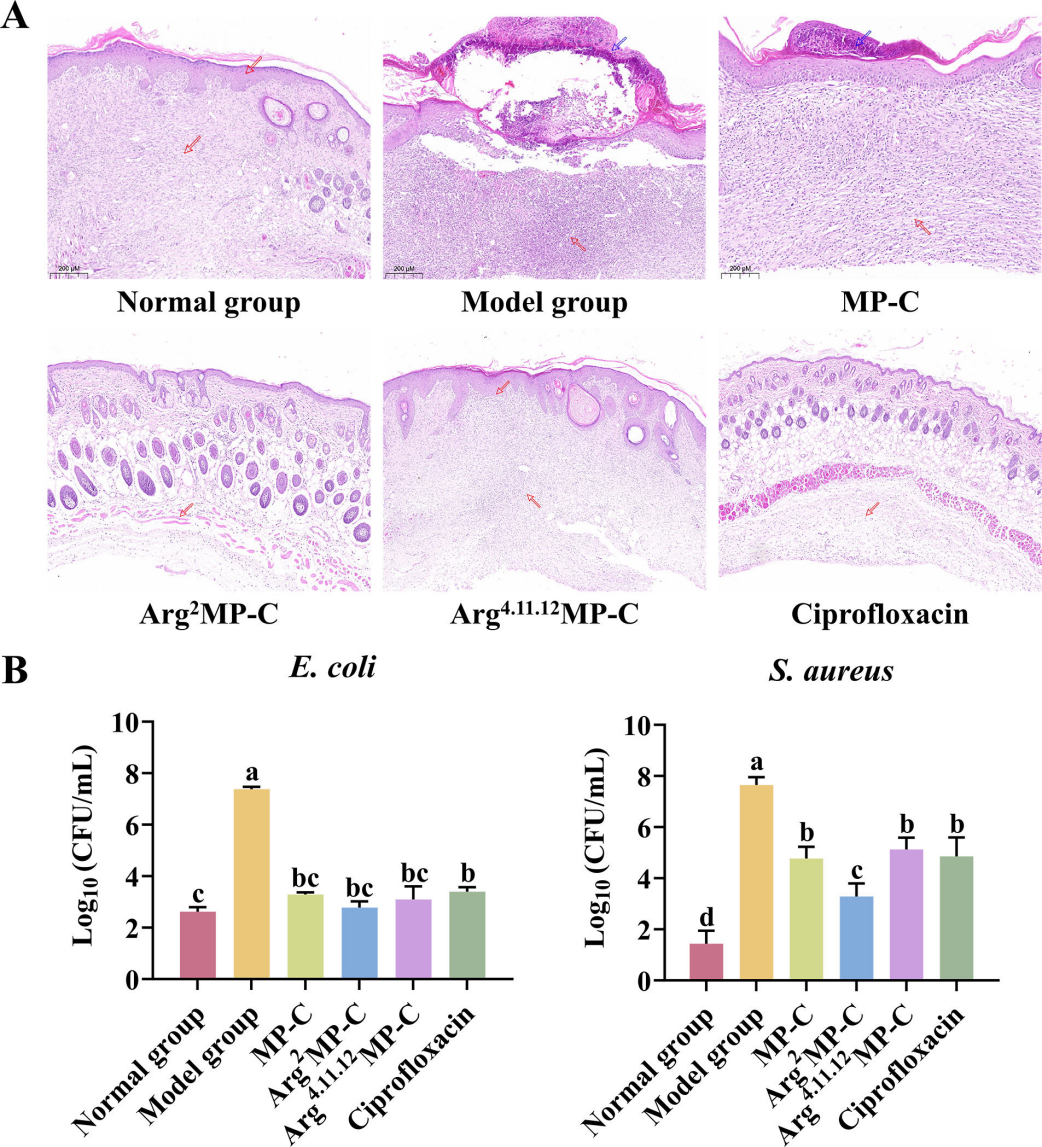
Histological analysis and bacterial clearance in infected wounds of mice. (**A**) HE staining of wound tissues. (**B**) Bacterial burden in wound tissues. Different lowercase letters (a, b, c, and d) above the bars indicate statistically significant differences between groups (*P* < 0.05), while the same letter indicates no significant difference.

Histopathological analysis with HE staining of the infected skin wounds from each group was presented in Fig. 8A. Evident erosion and loss (indicated by blue arrow) were observed in the epidermal layer, accompanied by substantial inflammatory cell infiltration in the subcutaneous tissue (indicated by red arrow), compared with the normal group. Compared with the model group, mice treated with MP-C, Arg^2^MP-C, and Arg^4.11.12^MP-C exhibited markedly reduced inflammatory cell infiltration (indicated by red arrow), a nearly restored upper epidermal structure, and the presence of new hair follicles and sebaceous glands. Among the MP-C, Arg^2^MP-C, and Arg^4.11.12^MP-C groups, the Arg^2^MP-C group displayed the most substantial epidermal regeneration, characterized by a well-structured epidermal layer, complete absence of eschar, and no signs of inflammation in the subcutaneous loose connective tissue.

### Skin tissue bacterial burden

The bacterial burden in the skin wound tissue of mice from each group is shown in Fig. 8B. Compared with the model group, all treatment groups significantly reduced the bacterial burden of both *E. coli* and *S. aureus*. Specifically, administration of MP-C and its derivatives reduced the *E. coli* burden to a level comparable to the normal group, exhibiting superior efficacy to the positive control ciprofloxacin group. Regarding *S. aureus*, although none of the treatments completely restored the bacterial burden to that of the normal group, Arg^2^MP-C still conferred a significantly greater reduction than the ciprofloxacin group. Collectively, these findings indicated that Arg^2^MP-C possessed the most potent therapeutic effect on mixed infections caused by *E. coli* and *S. aureus*.

## DISCUSSION

The skin serves as the primary barrier against microbial invasion. Following injury, the wound forms a complex environment containing microbes, necrotic tissue, and exudate, which is highly susceptible to polymicrobial infection and biofilm formation (33). The current clinical management of wound infections still relies heavily on antibiotics. However, the escalating global challenge of antimicrobial resistance severely undermines the efficacy of this approach. AMPs have attracted considerable research interest for their broad-spectrum activity, immunomodulatory capacity, and pro-healing properties (34). Therefore, this study aims to evaluate the efficacy of selected arginine-modified MP-C derivatives from a series of designed analogs against polymicrobial wound infections through both *in vitro* and *in vivo* models.

Through amino acid substitutions in MP-C, we obtained five derivatives (Pro^5^MP-C, Ile^6^MP-C, Val^10^MP-C, Arg^2^MP-C, and Arg^4.11.12^MP-C) and systematically analyzed their SARs. Among them, Pro^5^MP-C exhibits significantly reduced cytotoxicity and hemolytic activity while retaining its antibacterial efficacy. The substantial improvement in safety can be attributed to the proline-induced kink in the α-helical structure, which enhances selectivity for bacterial over mammalian membranes (35). The retained antibacterial activity of Pro^5^MP-C may result from increased overall hydrophobicity and a conformational shift toward a carpet-like mechanism, mediating bacterial membrane disruption via hydrophobic and electrostatic interactions (36, 37). Compared to MP-C, the antibacterial activities of Ile^6^MP-C and Val^10^MP-C were enhanced, which is likely attributable to increased hydrophobicity. Ile^6^MP-C exhibited stronger efficacy against *E. coli*, probably due to its elevated hydrophobicity, that may improve interaction with the lipopolysaccharide (LPS) in the outer membrane (OM) and facilitate penetration (38, 39). The valine substitution in Val^10^MP-C, which introduced a larger hydrophobic side chain to enhance membrane insertion and bilayer disruption, significantly improved its activity against *S. aureus* but concurrently increased non-selective hemolytic toxicity (40).

The arginine substitution in Arg^2^MP-C enhanced its net positive charge, yielding superior activity against *E. coli* probably from improved electrostatic interaction with LPS (41). Notably, this modification also reduced hemolysis slightly, as the bulky guanidinium group could sterically hinder peptide oligomerization and pore formation in neutral, cholesterol-rich erythrocyte membranes (42–44). In contrast to the single substitution in Arg^2^MP-C, replacing multiple lysines with arginines in Arg^4,11,12^MP-C markedly increased the density of guanidinium groups while maintaining the net positive charge, resulting in a dramatic enhancement of its activity against *S. aureus*. Compared to the lysine ammonium group, we propose that arginine can form stronger bidentate hydrogen bonds with phosphate head groups and enables cation-π interactions with lipid tails, promoting deeper membrane insertion and disruption, as evidenced in engineered peptides like WLBU2 (45). However, this potent, multivalent binding mechanism compromises selectivity, leading to the observed increase in cellular toxicity (46). Nevertheless, the anti-biofilm results show that the MP-C derivatives do improve biofilm inhibition (2-fold to 4-fold reduction in MBIC). However, the MBIC values themselves remain substantially higher than the corresponding MICs against planktonic bacteria, indicating that biofilm-specific tolerance remains a limiting factor.

MP-C and its derivatives showed generally stronger synergy against *S. aureus* than against *E. coli* in combination assays, potentially due to the permeability barrier of the gram-negative outer membrane (47). Unlike other derivatives, Arg^2^MP-C exhibited a universal additive effect against *E. coli* with all antibiotics, highlighting the benefit of its extra positive charge. Moreover, both arginine-substituted peptides synergized strongly with gentamicin and enrofloxacin against *S. aureus*, likely through membrane damage that enhances drug uptake for multi-target killing (48). These results underscore the potential of arginine-enriched derivatives as antibiotic adjuvants.

The exceptional antibacterial and synergistic profiles of Arg^2^MP-C and Arg^4,11,12^MP-C prompted a mechanistic investigation. Our studies showed that against *E. coli*, Arg^2^MP-C exhibited the most rapid disruption of the outer membrane at the early time point (16 μM). Moreover, it demonstrated the highest potency in disrupting the inner membrane at both the 5 and 25 min time points (32 μM). This behavior is attributed to the additional positive charge, which facilitates rapid outer membrane penetration and subsequent efficient inner membrane disruption (49, 50). Although SEM revealed that MP-C and Arg^4,11,12^MP-C induced more dramatic morphological damage, the functional data demonstrate that Arg^2^MP-C’s arginine-enhanced charge produces more immediately lethal membrane dysfunction. Against *S. aureus*, SYTOX Green assay indicated stronger membrane disruption by Arg^4,11,12^MP-C than by MP-C or Arg^2^MP-C. This finding is consistent with our SAR inference, suggesting that its higher guanidinium density may improve membrane targeting (45). Nevertheless, the observed membrane disruption appears insufficient to fully account for its potent antibacterial efficacy against *S. aureus*, implicating the involvement of other mechanisms.

Beyond membrane disruption, MP-C and its arginine derivatives may act through multi-target mechanisms. Both arginine-substituted peptides showed enhanced DNA binding to *S. aureus* compared to MP-C. Notably, Arg^4,11,12^MP-C exhibited even stronger binding to *E. coli* DNA than Arg^2^MP-C. This enhanced interaction is consistent with its greater guanidinium content, which probably promotes electrostatic and hydrogen-bonding contacts with the DNA backbone (51). Collectively, our findings demonstrate that the arginine-substituted derivatives exert their enhanced antibacterial effect through a multi-target mechanism, involving rapid membrane dysfunction followed by direct interaction with genomic DNA.

To evaluate the physiological relevance of our mechanistic findings, we examined the peptides in a murine wound model co-infected with *S. aureus* and *E. coli*. Arg^2^MP-C showed the strongest therapeutic effect, with accelerated wound closure, enhanced re-epithelialization, and minimal inflammatory infiltration. The superior healing and the lowest bacterial load in the Arg^2^MP-C group, indicating effective pathogen clearance, could be driven by its exceptional efficacy against *E. coli*, potentially through disrupting the polymicrobial community and fostering a healing-conducive microenvironment (52). Although Arg^4,11,12^MP-C showed stronger direct activity against *S. aureus in vitro* and shared the same MBIC with Arg^2^MP-C, the latter led to a more pronounced reduction in *S. aureus* burden and was equally effective against *E. coli in vivo*. This suggests that additional factors such as the ability to maintain proteolytic stability or host immune interactions may critically influence antibacterial efficacy in complex infection environments (42, 53). Consistent with this, the modest 2-fold to 4-fold improvement in biofilm inhibition does not fully capture the *in vivo* advantage of Arg^2^MP-C. One possibility is that its rapid membrane disruption could help lower bacterial load before biofilm establishment. In addition, its strong cationic charge might facilitate interactions with wound matrix or epithelial cells (54, 55). Furthermore, Mastoparan-derived peptides are known to have mild anti-inflammatory properties, which could also contribute to tissue repair (56).

In summary, our systematic investigation of MP-C derivatives reveals distinct functional benefits conferred by specific amino acid substitutions. Pro^5^MP-C exhibited markedly enhanced selectivity, while hydrophobic variants (Ile^6^MP-C and Val^10^MP-C) displayed increased antibacterial potency. Arginine-substituted peptides further conferred a multi-target mechanism involving membrane disruption and DNA binding. Notably, Arg^2^MP-C, which possessed the strongest activity against *E. coli in vitro*, demonstrated comprehensive *in vivo* efficacy that surpassed even derivatives with superior anti-staphylococcal profiles. These findings affirm that *in vivo* performance is essential in evaluating antimicrobial peptides and establish Arg^2^MP-C as a promising candidate for treating complex wound infections.

## SUPPLEMENTAL MATERIAL

Tables S1 and S2, and Figures S1 to S3 spectrum.04219-25-s0001.docx Combined antibacterial results, peptides identification, *E. coli* inner membrane disruption.

ASM does not own the copyrights to Supplemental Material that may be linked to, or accessed through, an article. The authors have granted ASM a non-exclusive, world-wide license to publish the Supplemental Material files. Please contact the corresponding author directly for reuse.
