# Supplementary material for: Arginine-substituted Mastoparan-C derivatives combat dual bacterial pathogens: in vitro mechanistic insights and in vivo efficacy in polymicrobial wounds
Source: Microbiol Spectr. 2026 Jun 29;14(8):e04219-25. doi: 10.1128/spectrum.04219-25 (PMC13436381; doi:10.1128/spectrum.04219-25)
Supplement: Supplementary file 1 [file spectrum.04219-25-s0001.docx]

**Arginine-substituted Mastoparan-C derivatives combat dual bacterial pathogens: *in vitro* mechanistic insights and *in vivo* efficacy in polymicrobial wounds**

Luyao Zhang ^a,#^, Anqi Huang ^a,#^, Mingyu Lu ^a^, Jingjing Wang ^a^, Yingyu Wang ^a^, Fei Mo ^a,*^ and Mingchun Liu ^a^

**Affiliations:**

^a^ College of Animal Science and Medicine, Shenyang Agricultural University, Shenyang, 110866, China.

^#^ Luyao Zhang, and Anqi Huang contributed equally to this article.

* Corresponding author.

Address correspondence to Fei Mo, mf07@syau.edu.cn.

**Supplementary materials**

**Table S1 Combined antibacterial results of MP-C and its derivatives with four conventional antibiotics**

| *E. coli* | | |
| --- | --- | --- |
| Antimicrobial peptide+antibiotic | FIC index | Combined effect |
| MP-C+gentamicin | 0.5625 | Additive |
| MP-C+tylosin | 0.625 | Additive |
| MP-C+enrofloxacin | 0.5625 | Additive |
| MP-C+ceftiofur sodium | 0.75 | Additive |
| Pro^5^MP-C+gentamicin | 0.5625 | Additive |
| Pro^5^MP-C+tylosin | 0.625 | Additive |
| Pro^5^MP-C+enrofloxacin | 1.0625 | Indifferent |
| Pro^5^MP-C+ceftiofur sodium | 0.5625 | Additive |
| Val^10^MP-C+gentamicin | 1.0625 | Indifferent |
| Val^10^MP-C+tylosin | 1 | Indifferent |
| Val^10^MP-C+enrofloxacin | 0.5625 | Additive |
| Val^10^MP-C+ceftiofur sodium | 0.5625 | Additive |
| Ile^6^MP-C+gentamicin | 1.5 | Indifferent |
| Ile^6^MP-C+tylosin | 2 | Indifferent |
| Ile^6^MP-C+enrofloxacin | 1 | Indifferent |
| Ile^6^MP-C+ceftiofur sodium | 1.5 | Indifferent |
| Arg^2^MP-C+gentamicin | 0.5625 | Additive |
| Arg^2^MP-C+tylosin | 0.625 | Additive |
| Arg^2^MP-C+enrofloxacin | 0.5625 | Additive |
| Arg^2^MP-C+ceftiofur sodium | 0.5625 | Additive |
| Arg^4.11.12^MP-C+gentamicin | 0.5625 | Additive |
| Arg^4.11.12^MP-C+tylosin | 0.625 | Additive |
| Arg^4.11.12^MP-C+enrofloxacin | 1.0625 | Indifferent |
| Arg^4.11.12^MP-C+ceftiofur sodium | 1 | Indifferent |
| *S. aureus* | | |
| Antimicrobial peptide+antibiotic | FIC index | Combined effect |
| MP-C+gentamicin | 0.5625 | Additive |
| MP-C+tylosin | 1.0625 | Indifferent |
| MP-C+enrofloxacin | 0.3125 | Synergistic |
| MP-C+ceftiofur sodium | 0.5625 | Additive |
| Pro^5^MP-C+gentamicin | 0.5625 | Additive |
| Pro^5^MP-C+tylosin | 0.5625 | Additive |
| Pro^5^MP-C+enrofloxacin | 0.5625 | Additive |
| Pro^5^MP-C+ceftiofur sodium | 0.5625 | Additive |
| Val^10^MP-C+gentamicin | 1 | Indifferent |
| Val^10^MP-C+tylosin | 1.0625 | Indifferent |
| Val^10^MP-C+enrofloxacin | 1.0625 | Indifferent |
| Val^10^MP-C+ceftiofur sodium | 0.5625 | Additive |
| Ile^6^MP-C+gentamicin | 1.0625 | Indifferent |
| Ile^6^MP-C+tylosin | 0.125 | Synergistic |
| Ile^6^MP-C+enrofloxacin | 0.5625 | Additive |
| Ile^6^MP-C+ceftiofur sodium | 0.5625 | Additive |
| Arg^2^MP-C+gentamicin | 0.3125 | Synergistic |
| Arg^2^MP-C+tylosin | 1.0625 | Indifferent |
| Arg^2^MP-C+enrofloxacin | 0.3125 | Synergistic |
| Arg^2^MP-C+ceftiofur sodium | 0.5625 | Additive |
| Arg^4.11.12^MP-C+gentamicin | 0.3125 | Synergistic |
| Arg^4.11.12^MP-C+tylosin | 1.0625 | Indifferent |
| Arg^4.11.12^MP-C+enrofloxacin | 0.3125 | Synergistic |
| Arg^4.11.12^MP-C+ceftiofur sodium | 0.5625 | Additive |

**Table S2 Theoretical and observed masses of peptides determined by mass spectrometry**

| **Peptides** | **Theoretical Mass (Da)** | **Observed Mass (Da)** | **Error (Da)** | **Observed [M+2H] ²⁺ (m/z)** | **Observed [M+3H] ³⁺ (m/z)** |
| --- | --- | --- | --- | --- | --- |
| MP-C | 1506.96 | 1507.20 | ±0.24 | 754.70 | 503.40 |
| Pro^5^MP-C | 1533.00 | 1533.12 | ±0.12 | 767.56 | 512.05 |
| Val^10^MP-C | 1535.01 | 1535.30 | ±0.29 | 768.65 | 512.75 |
| Ile^6^MP-C | 1506.96 | 1507.20 | ±0.24 | 754.60 | 503.55 |
| Arg^2^MP-C | 1549.04 | 1548.80 | ±0.24 | 775.40 | 517.25 |
| Arg^4.11.12^MP-C | 1591.00 | 1591.50 | ±0.50 | 796.30 | 531.50 |

Fig. S1 Mass spectrometry identification results of MP-C and its derivatives.

(A~F): MP-C；Pro^5^MP-C；Ile^6^MP-C；Val^10^MP-C；Arg^2^MP-C；Arg^4.11.12^MP-C.

Fig. S2 Chromatographic identification results of MP-C and its derivatives.

(A~F): MP-C；Pro^5^MP-C；Ile^6^MP-C；Val^10^MP-C；Arg^2^MP-C；Arg^4.11.12^MP-C.

Fig. S3 *E. coli* inner membrane disruption by MP-C, Arg^2^MP-C and Arg^4.11.12^MP-C at 32 μM at 5 and 25 min. ** indicates highly significant differences (*p*<0.01).
